# Mexican-origin parent and child reported neighborhood factors and youth substance use

**DOI:** 10.3389/fpsyt.2023.1241002

**Published:** 2023-12-01

**Authors:** Jenny Zhen-Duan, Devin E. Banks, Caroline Ferreira, Lulu Zhang, Kristin Valentino, Margarita Alegría

**Affiliations:** ^1^Disparities Research Unit, Massachusetts General Hospital, Boston, MA, United States; ^2^Department of Psychiatry, Harvard Medical School, Boston, MA, United States; ^3^Department of Psychological Sciences, University of Missouri-St. Louis, St. Louis, MO, United States; ^4^Department of Psychiatry, Yale School of Medicine, New Haven, CT, United States; ^5^Department of Psychology, University of Notre Dame, Notre Dame, IN, United States; ^6^Department of Medicine, Harvard Medical School, Boston, MO, United States

**Keywords:** neighborhood factors, substance use, Mexican, Latinx, parent and child

## Abstract

**Background:**

Structural oppression affects health behaviors through residence in suboptimal neighborhoods and exposure to community violence. Youth and parents report perceptions of neighborhood factors that can affect youth substance use behaviors. Given that Latinx youth report higher levels of perceived community violence than other racial and ethnic groups, it is imperative to examine how youth- and parent-perceived neighborhood-level factors may relate to youth substance use.

**Methods:**

Data were collected using clinical interviews with family triads (fathers, mothers, and youth) and parent–child dyads (father or mother and youth) enrolled in the *Seguimos Avanzando* study of 344 Mexican-origin families in Indiana. Neighborhood measures, including perceptions of exposure to violence, neighborhood characteristics, and neighborhood collective efficacy, were included in parent and youth surveys. Self-report measures for past year alcohol and drug use were included in the youth survey only. *T*-tests were conducted to estimate differences in neighborhood reports among the sample triads. A series of linear regression models were used to estimate the associations between youth-, mother-, and father-reported perceptions of neighborhood factors and youth substance use.

**Results:**

Preliminary results indicate that fathers reported higher levels of exposure to violence than mothers [*t*(163) = 2.33, *p* = 0.02] and youth [*t*(173) = 3.61, *p* < 0.001]. Youth reported lower negative neighborhood characteristics than mothers [*t*(329) = 6.43, *p* < 0.001] and fathers [*t*(169) = 3.73, *p* < 0.001]. Youth reported significantly better neighborhood collective efficacy than mothers [*t*(296) = 3.14, *p* = 0.002], but not statistically different from fathers. Results from the primary analysis showed that youth exposure to violence was positively associated with youth substance use (*b* = 0.24, SE = 0.06, *p* < 0.0001), but the youth’s neighborhood characteristics and collective efficacy were not significantly associated with youth substance use. None of the parent-reported neighborhood variables were associated with youth substance use.

**Conclusion:**

The discrepant findings between parent and youth reports of perceived neighborhood characteristics and substance use have important implications for researchers and community stakeholders, and for developing targeted interventions and prevention strategies. Our study highlights the need to address youth experience of community violence and to prioritize creating safe and inclusive neighborhood environments. Potential strategies include improving community resources, strengthening social support networks, promoting open communication about neighborhood risks, and fostering collaborative efforts to address substance use behaviors.

## Introduction

The Substance Abuse and Mental Health Services Administration (SAMHSA) estimated that in 2021, a total of 46.3 million (16.5%) individuals 12 and older experienced a substance use disorder (SUD) in the past year. This includes 29.5 million people with alcohol use disorder, 24 million with drug use disorder, and 7.3 million with both drug and alcohol use disorders. Among those with an SUD in the past year, 15.7% identified themselves as Hispanic or Latinx (1). The current literature on substance use risk has mostly examined individual-level factors among Latinx youth, including family (2–4) and cultural factors (5), and socio-emotional problems (6). Less research examines how perceived neighborhood factors play a role in the initiation of use and increased risk of substance use. Drawing from structural oppression and health models (7) along with social disorganization theory (8), the current paper addresses this gap in the literature by examining how perceived neighborhood factors, as reported by fathers, mothers, and youth, might be associated with substance use among Mexican-origin youth in the United States.

### Theoretical frameworks

Structural oppression and structural racism are operationalized as structures or practices that foster discrimination and loss of opportunities in minoritized populations through laws, policies, practices, and ideologies (7, 9). Structural oppression affects health and substance use behaviors, directly and indirectly, through residing in suboptimal neighborhood conditions and exposure to community violence (10). These structural drivers then lead to social disorganization and low levels of social control within a community that can lead to delinquent behaviors (e.g., gangs) and informal community control (8). For Latinx people, structural oppression can manifest in economic hardship, language dominance, and cultural differences, leading to residential segregation into more impoverished and isolated neighborhoods (11, 12). In fact, approximately 50% of Latinx people in the United States (U.S.) would have to relocate and move elsewhere to end segregation, a figure that has changed little since the 1980s (11, 12). In addition to poverty, Latinx neighborhoods in the U.S. tend to be characterized by poor education access and high levels of stressors, including self-reported physical neighborhood decay, social disorder, and victimization (12, 13). Accordingly, Latinx youth report exposure to some of the highest rates of community violence and social disorganization compared to other racial and ethnic groups (14), pointing to the importance of understanding how perceptions of neighborhood-level factors can influence the pathways of risk and resilience for Latinx youth substance use. While more objective measures of neighborhood characteristics (e.g., crime rates) may impact Latinx health, less is known on ways perceptions of neighborhood characteristics affect substance use behaviors. Objective measures of the neighborhood only tell part of the story, with some evidence that subjective reports of neighborhoods may play a more important role in explaining outcomes (15). Subjective perceptions of neighborhood factors are also important to study among household members because each may have different perspectives on how neighborhood factors uniquely contribute to substance use risks.

### Neighborhood-level factors and their relation to substance use

Exposure to “socially toxic environments” during childhood, including neighborhoods characterized by housing and economic instability, community socioeconomic disadvantage, and neighborhood violence and crime, increase vulnerability to substance use initiation and disorder (16). Neighborhood factors influence substance use at many ecological levels, including individual, interpersonal, community, and policy levels. For instance, at the individual biological level, exposure to neighborhood stress and trauma during childhood has been linked to substance use vulnerability via dysregulation of neurological reward pathways involved in risk-taking and activation of the physiological stress response, which is linked to craving (16). Neighborhood factors and substance use have also been linked through community mechanisms like increased availability and exposure to substances commercially and illicitly (17–19). Accordingly, among a sample of 9th-grade adolescents, neighborhood socioeconomic disadvantage and neighborhood substance use exposure were associated with greater use of substances after controlling for school, peer, family, and individual socioeconomic factors (20). Despite this evidence, several studies suggest the relation between neighborhood-level socioeconomic disadvantage and substance use is inconsistent, particularly among youth population samples (17, 21). To clarify these mixed findings, it is essential to consider additional neighborhood factors comprising a socially toxic environment, such as exposure to violence and lack of community cohesion and opportunity, as posited within social disorganization theories (8).

Given the strong link between chronic stressful experiences during childhood (e.g., adverse childhood experiences) and substance use vulnerability, it is not surprising that exposure to violence in neighborhoods is a significant risk factor for substance use and other negative behaviors (2, 22). Youth who report more exposure to community violence (e.g., witnessing violence) also report greater use of alcohol and other substances (23, 24). Latinx youth experience higher rates of community violence compared to other racial/ethnic groups, making this a particularly relevant aspect of the environment (14). Specifically, among Latinx youth, exposure to community violence is associated with greater frequency of individual substance use (25, 26) and polysubstance use (27). Latinx youth who report more exposure to or worry about gangs in their community also report greater frequency of substance use (27, 28). A prospective study also found community violence exposure predicted early alcohol initiation among Puerto Rican adolescents (29). Despite this evidence, when community violence is measured at the neighborhood level—that is, via rates of crime from local or federal administrative data—it does not demonstrate associations with individual substance use among Latinx youth (30, 31), pointing to the importance of perceptions of a socially toxic environment.

Other community-related perceptions associated with adolescent substance use include negative neighborhood characteristics representing what is often called “social disorder” or “neighborhood disorganization” (8). Negative neighborhood characteristics include individuals’ proximity to gangs and gang-associated violence, the drug trade, petty crime, and negative aspects of the built environment, such as vacancies and deteriorating building structures (32, 33). Youth who live in increasingly deteriorating or disorganized environments are also more likely to report substance use and related consequences in emerging adulthood than those in environments with low or decreasing disorganization (34, 35). Given that neighborhood disadvantage can be assessed through a wide range of social disorder observations, measurements of negative neighborhood characteristics can be a powerful community-level predictor of adolescent substance use according to nationally representative data (36). For example, one study among Latinx and African American youth found that among several neighborhood variables, only parent-perceived social disorder during pre-adolescence predicted substance use initiation by adolescence (31), highlighting that parents’ neighborhood perspectives and perceptions can also impact youth substance use behaviors.

Inversely related to neighborhood disorganization is neighborhood collective efficacy, which could serve as a buffer against substance use risk. Neighborhood collective efficacy, which refers to a neighborhood’s social capital or social cohesion (37), is considered a protective factor against substance use and encompasses the extent to which neighbors support and trust each other and work together to maintain social order. Higher levels of neighborhood collective efficacy have been associated with lower levels of substance use and other negative outcomes among youth (38). These factors are associated with lower substance use among adolescents (36, 39), perhaps because they represent greater social bonds and monitoring associated with communalism. For example, one longitudinal study found that lower parent-reported neighborhood collective efficacy (specifically, social cohesion) at age 12 was associated with greater adolescent substance use at age 17 (40). However, mixed and null findings on the impact of collective efficacy on adolescents’ substance use have also been noted (35, 41). Among Latinx adolescents, findings have been mixed even among aspects of neighborhood collective efficacy. For example, one study found that parents-perceived neighborhood social cohesion, but not neighborhood social control, predicted adolescent alcohol use (42).

### Parent and youths’ perceptions of neighborhood-level factors and substance use

Parental perceptions of neighborhood factors may be significantly related to youth adjustment (43) and their likelihood of engaging in substance use (40). Parents residing in neighborhood with high drug availability may alter their parenting practices to shield their children from harm risks, including setting stricter rules and monitoring their children’s activities more closely to protect them from substance use initiation (44, 45). Conversely, positive perceptions of the neighborhood, including a positive sense of community (46, 47) and prosocial peer behavior (48), have been linked to lower levels of substance use among youth. When young individuals perceive their neighborhoods as safe and supportive, they may be less likely to use substances to cope (49). Positive neighborhood characteristics can promote a sense of belonging, social cohesion, and opportunities for positive engagement, which are protective factors for mental health (50). The relations between neighborhood characteristics and substance use are not solely determined by objective measures, but are also influenced by individuals’ perceptions and experiences in the neighborhood (51, 52). For example, both fathers’ and mothers’ perceptions of neighborhood quality directly and indirectly affected Latino boys’ substance use behaviors (either increased or decreased the risk). Youth’s own perception of neighborhood quality was associated with decreased substance use risk (53). Individuals in the same neighborhood may have varying perceptions of the same characteristics based on their circumstances, interactions, and social networks. Thus, it is crucial to consider the subjective experiences of both parents and youth when examining the impact of neighborhood factors on substance use.

## Current study

Examining the perceptions and reports of parents and youth regarding multiple neighborhood factors can provide a more comprehensive understanding of how these factors may influence Latinx youth substance use. This cross-sectional study aims to address the dearth of subjective neighborhood perceptions by multiple reporters, by examining parent- and youth-reported neighborhood perspectives and their link to substance use, focusing on a Latinx sample. We examined how Mexican-origin fathers’, mothers’, and youth’s reports of neighborhood community violence, negative neighborhood characteristics, and neighborhood collective efficacy influenced youth substance use. Understanding the shared or diverging experiences and perspectives of both parents and youth, between perceptions of neighborhood factors and their impact on substance use, is crucial for informing targeted prevention and intervention strategies to reduce substance use among Latinx youth. Our key hypotheses were that greater parent- and youth-reported community violence, negative neighborhood characteristics, and less neighborhood collective efficacy would be associated with increased likelihood of youth-reported substance use.

## Methods

### Participants and procedures

The *Seguimos Avanzando* project is a prospective study of 344 Mexican-origin families in Northern Indiana aimed at examining mechanisms linking discrimination and health outcomes. *Seguimos Avanzando* is unique in that it is a longitudinal study emphasizing the recruitment of family triads (e.g., fathers, mothers, and youth). It also included parent–child dyads (e.g., father or mother, and youth) if only one parent was able to, or willing to, participate. To be eligible for this study, participating family members (i.e., parents/caregivers and youth) had to identify as Mexican-origin, and youth had to be between 12 and 15 years old at baseline. Main caregivers included biological parents, same-sex parents, stepparents, guardians, and extended family members [see Alegría et al. (54) for additional study information]. Families were ineligible to participate if they had participated in the pilot study (55) or if parents reported a youth learning or developmental disability. The final baseline sample included *N* = 344 families, of which *N* = 162 were triads and *N* = 182 were dyads.

Families were recruited with the support of community partners who had helped recruit the pilot study participants and through other community-based organizations (e.g., churches, nonprofits) and community events targeted towards Latinx and Mexican American families. Potential participants completed eligibility assessments in person or over the phone. Parents/caregivers signed consent forms for their participation and the youth’s participation while the youth signed assent forms. Bilingual research assistants conducted the interviews in the families’ preferred locations, such as their homes or in community-based centers, or via videoconferencing (i.e., Zoom). Most interviews were conducted one on one by separate research assistants located in separate and private rooms. In some cases where one family member was unavailable to be interviewed simultaneously, interviewers coordinated a different visit to complete the missing interviews. The University of Notre Dame approved this study with ceded reviews from other participating institutions.

### Measures

Neighborhood measures, including exposure to violence, neighborhood characteristics, and neighborhood collective efficacy, were included in both parent and youth surveys. In contrast, a self-report measure for past year alcohol and drug use was included in the youth survey only.

### Neighborhood factors

#### Exposure to violence in the neighborhood

An adapted version of the Exposure to Violence Scale (56) was used to assess the degree of violence exposure in the neighborhood. The 10-item survey measures how many different threats people face in their communities (e.g., being chased, arrested, experiencing and/or witnessing a physical assault, or witnessing suicide or homicide). Respondents indicated whether they either directly experienced (0 *does not apply* or 1 *apply*) or witnessed (0 *does not apply* or 1 *apply*) each violence exposure. A third response option assessing for vicarious exposure to violence (i.e., whether they know that a certain violence experience happened to someone else, but they did not personally witness it) was excluded from the response options for this study, in order to only assess individuals’ direct exposure to violence. The final score was calculated as the sum of all affirmative responses (either directly experienced or witnessed), which ranged from 0 to 20, with higher scores indicating greater direct exposure to violence.

#### Negative neighborhood characteristics

The Neighborhood Characteristics scale assesses participants’ perceptions of neighborhood problems (32) and has been previously used in studies including a Puerto Rican youth sample (57). This 12-item survey assesses neighborhood problems such as unemployment rates, delinquency, abandoned houses or buildings, drug sales, and lack of community connectedness. Ten questions asked participants to rate “how much of a problem” these were in their community, including *0. not a problem*, *1. a problem but not too serious*, or *2. a serious problem*. Two final items asked about perceptions about community connectedness and belonging, with responses endorsing *0. almost always true* and *1. almost never true*. The total score is calculated by the sum of all the first ten 3-scale items and the last two dichotomous items, which ranged from 0 to 22, with higher scores indicating worse neighborhood characteristics.

#### Neighborhood collective efficacy

The Collective Efficacy Scale (37) is an 8-item scale that measures social cohesion and trust. Responses for the first seven questions use a 4-point scale *(1 = very true; 4 = not at all true)* to rate the statements such as, “People in your neighborhood can be trusted.” The score is calculated by the sum of the seven items, with a higher score indicating better collective efficacy (range = 7–28). A final question asks participants to name the number of neighbors known by name, which was not included in the composite score.

### Youth substance use

The CRAFFT Questionnaire 2.1, originally developed by the Center for Adolescent Substance Abuse Research (58), assessed past-year alcohol and drug use. The original 9-item health screening questionnaire consists of questions developed to identify current and future problematic substance use, substance-related risks, and substance use disorder among youth ages 12–21. The CRAFFT 2.1 version was used, in which the first four questions asked youth to report the number of days they engaged in alcohol, marijuana, and other drug use in the past year and whether they rode in a car with someone who had used alcohol or drugs (1 *yes* and 0 *no*). If the youth answered at least 1 day of using any of the three substances, then they were asked five additional questions on problems due to use and risk related to use (e.g., use alcohol or drugs to relax or while alone, forget things due to own use) with the response options of 1 *yes* and 0 *no* (59). The total score was calculated using the sum of six dichotomized items, ranging from 0 to 6.

### Additional baseline demographic characteristics

Baseline youth demographic characteristics include age (12–16 years old), gender (male, female, non-binary/third gender), birthplace (United States or Mexico) family structure (two-parent family, other), and survey mode (virtual/phone, in person). Baseline mother and father demographic characteristics included age (mother, 20–61 years old; father, 24–73 years old), gender, birthplace, and family income in a year (less than $30,000, $30,000 – $69,000, $70,000 or more).

### Data analysis

All analyses were conducted in STATA 17.0. We begin by presenting the distribution of all study measures (youth substance use, neighborhood factors, and additional baseline demographic characteristics) from the youth, mother, and father reports. Continuous variables’ descriptive information was presented as mean and standard deviation (SD) or mean, median, and range. Descriptive information for categorical variables was presented as frequencies and percentages. A *t*-test was performed for examining the differences in neighborhood factors between reports from youth, mothers, and fathers. We then examined the correlation among all study variables from the youth, mother, and father. Afterward, we examined whether youth-, mother-, and father-reported neighborhood factors were associated with youth-reported substance use. These associations were estimated using linear regression models. Missing data from youth reports ranged from 0.3% (*N* = 1) for exposure to violence to 10.8% (*N* = 37) for neighborhood collective efficacy. Missing data from mother reports ranged from 0.3% (*N* = 1) for neighborhood collective efficacy to 1.2% (*N* = 4) for negative neighborhood characteristics. Missing data from father reports ranged from 1.1% (*N* = 2) for exposure to violence to 3.4% (*N* = 6) for negative neighborhood characteristics. As shown elsewhere (54) the missing data mechanism within the current study sample can be assumed to be missing at random. Under this assumption, we fitted our linear regression models via Full Information Maximum Likelihood to handle missing data. Three separate models were estimated, one using youth reports, one using mother reports, and one using father reports. All models adjusted for baseline youth age, youth gender (male vs. other), youth-reported family structure (two-parent family vs. other), and youth survey mode (in-person vs. virtual/phone). Youth gender was included as male vs. other, given that very few youth self-identified as non-binary/third gender (N = 8). Further, the distribution of the outcome variable was skewed toward zero (78.6% of youth reported never used substances). Given that recent evidence highlight that common transformation for skewed data (e.g., data with high counts of zero) does not perform well (60), we conducted a sensitivity analysis to compare the association between neighborhood factors and youth substance use, using Poisson models instead of linear models to account for the skewness of our data (see Supplementary Table S1). As expected, none of the results were different from the linear regression models. Thus, we present the linear regression results with untransformed data.

## Results

Table 1 illustrates the sample demographic information. A total of 344 youth reports were collected. The mean age was 13.5 and 51.7% of youth were male. The average youth age was 13, and in terms of gender, most identified as male, followed by female, and only three youth identified as non-binary/third gender. Almost all youth were born in the US (93.0%), and 98.3% self-identified their ethnicity as Mexican or Mexican American. The outcome variable, the average CRAFFT score, includes 250 (78.6%) youth who reported no substance use. The overall mean score was 0.3, with a median of 0, and ranged from 0 to 5. 80.5% of youth participants reported residing in a two-parent family. Among the 335 mother reports and 176 father reports collected, most parents reported being born in Mexico (mother = 92.5%, father = 94.9%). More than half of the families reported annual income from $30,000 to $69,000 (mother = 61.5%, father = 63.6%).

**Table 1:** Baseline sociodemographic information reported by youth, mothers, and fathers.

	Youth report	Mother report	Father report
	*N* = 344	*N* = 335	*N* = 176
**Outcome variable, youth reported**			
Substance Use, CRAFFT score, mean (median) [range]	0.3 (0.0) [0–5]	NA	NA
**Explanatory variables**			
Negative neighborhood characteristics, *mean (SD)*	11.0 (16.1)	5.4 (5.0)	5.1 (5.7)
Neighborhood collective efficacy, *mean (SD)*	19.7 (4.6)	18.8 (5.1)	20.3 (4.8)
Exposure to violence, *mean (SD)*	1.7 (2.6)	2.3 (2.7)	3.1 (3.6)
**Sociodemographic characteristics**			
**Age, *mean (SD)***	13.5 (1.1)	41.4 (6.4)	44.0 (7.8)
**Gender, *n (%)***			
Male	178 (51.7%)	0 (0.0%)	176 (100.0%)
Female	158 (45.9%)	333 (99.4%)	0 (0.0%)
Non-binary/third gender	8 (2.3%)	2 (0.6%)	0 (0.0%)
**Birthplace, *n (%)***			
US	320 (93.0%)	24 (7.2%)	9 (5.1%)
Mexico	23 (6.7%)	310 (92.5%)	167 (94.9%)
Other^a^	0 (0.0%)	1 (0.3%)	0 (0.0%)
Missing	1 (0.3%)	0 (0.0%)	0 (0.0%)
**Family income in a year, *n (%)***			
Less than $30,000	NA	94 (28.1%)	30 (17.0%)
$30,000 - $69,000	NA	206 (61.5%)	112 (63.6%)
$70,000 or more	NA	35 (10.4%)	34 (19.3%)
**Youth report, family structure, *n (%)***			
Two-parent family	277 (80.5%)	NA	NA
Other	67 (19.5%)	NA	NA
**Youth survey mode, *n (%)***			
Virtual/Phone	87 (25.3%)	NA	NA
In person	257 (74.7%)	NA	NA

^a^The mother selected other category and specified as born in Honduras.

With regards to neighborhood factors, fathers reported higher levels of exposure to violence than mothers [*t*(163) = 2.35, *p* = 0.02] and youth [*t*(173) = 3.61, *p* < 0.001]. Youth reported lower negative neighborhood characteristics than mothers [*t*(329) = 6.43, *p* < 0.001] and fathers [*t*(169) = 3.72, *p* < 0.001]. In addition, youth reported significantly better neighborhood collective efficacy than mothers [*t*(296) = 3.14, *p* = 0.002], but not statistically different from fathers. Fathers reported better neighborhood collective efficacy than mothers [*t*(163) = 2.38, *p* = 0.02]. No statistically significant mean differences were observed between fathers and mothers in negative neighborhood characteristics.

The correlation table in Table 2 indicates that none of the paired variables from the youth, mother, and father reports demonstrated a strong correlation (*r* > 0.7). A significant positive correlation was observed between youth-reported exposure to violence and youth substance use (*r* = 0.23, *p* < 0.05), suggesting youth with higher substance use tend to report greater exposure to violence or vice versa. The relation between neighborhood collective efficacy reported by the youth with the reports by the parents (mother, *r* = 0.41, *p* < 0.05; father, *r* = 0.38, *p* < 0.05) are positively correlated.

**Table 2:** Correlation table.

	1	2	3	4	5	6	7	8	9	10	11	12	13	14
**Outcome**														
1. Youth CRAFFT	1													
**Youth**														
2. Negative neighborhood characteristic	0.05	1												
3. Neighborhood collective efficacy	−0.05	−0.09	1											
4. Exposure to violence	0.23*	−0.10	−0.11	1										
**Mother**														
5. Negative neighborhood characteristic	0.00	0.16*	−0.22*	0.03	1									
6. Neighborhood collective efficacy	0.01	−0.09	0.41*	−0.08	−0.41*	1								
7. Exposure to Violence	0.00	−0.02	−0.18*	0.06	0.19*	−0.17*	1							
**Father**														
8. Negative Neighborhood Characteristic	−0.06	−0.07	−0.12	0.11	0.30*	−0.28*	0.02	1						
9. Neighborhood Collective Efficacy	0.04	−0.02	0.38*	−0.16*	−0.27*	0.32*	−0.06	−0.30*	1					
10. Exposure to Violence	−0.07	−0.12	0.09	−0.04	−0.02	−0.04	0.09	0.13	0.00	1				
**Covariates**														
11. Age	0.15*	−0.03	0.03	0.10	0.05	0.04	0.01	0.06	−0.05	−0.13	1			
12. Male	−0.12*	−0.08	−0.04	0.06	0.00	−0.02	0.00	−0.09	0.06	−0.03	0.01	1		
13. Family Structure	−0.12*	−0.09	0.09	−0.06	−0.17*	0.09	−0.15*	0.01	0.11	−0.12	0.01	0.04	1	
14. Survey Mode	0.03	−0.05	−0.01	0.01	0.09	−0.03	0.09	0.11	0.05	−0.02	−0.01	−0.07	−0.17	1

*P < 0.05.

The presented linear regression models (Table 3) examined the association between youth substance use and exposure to violence, neighborhood characteristics, and neighborhood collective efficacy, as well as youth, mother, and father independent reports. The results, shown in standardized scores, indicate that youth’s reported exposure to violence was positively associated with youth substance use (*b* = 0.24, SE = 0.05, *p* < 0.0001), suggesting that higher levels of exposure are associated with increased substance use. No significant associations were observed between mother- or father-reported neighborhood factors and youth substance use.

**Table 3:** Association between youth CRAFFT score and negative neighborhood characteristics, neighborhood collective efficacy, and exposure to violence from youth, mother and father report.

	Coeff.				Std. err.	95% CI	*P*
	Youth
Negative neighborhood characteristics	0.101	0.06	[−0.02, 0.22]	0.112
Neighborhood collective efficacy	−0.021	0.06	[−0.13, 0.09]	0.713
Exposure to violence	0.244	0.05	[0.14, 0.35]	< 0.001
	Mother			
Negative neighborhood characteristics	−0.017	0.06	[−0.13, 0.10]	0.776
Neighborhood collective efficacy	0.007	0.06	[−0.11, 0.13]	0.901
Exposure to violence	−0.017	0.06	[−0.13, 0.09]	0.754
	Father			
Negative neighborhood characteristics	−0.067	0.10	[−0.26, 0.12]	0.489
Neighborhood collective efficacy	0.053	0.09	[−0.13, 0.24]	0.577
Exposure to violence	−0.067	0.09	[−0.25, 0.11]	0.467

## Discussion

Drawing from structural oppression and health frameworks (7) as well as social disorganization theory (8), this study aimed to estimate the degree to which parent- and child-reported neighborhood factors impacted youth substance use. Three main findings emerged. First, youth-reported exposure to violence was associated with increased substance use. Second, negative neighborhood characteristics and neighborhood collective efficacy were not associated with substance use. Third, none of the parent reports of neighborhood factors, including parents’ reports of exposure to violence, were associated with substance use among youth.

Youth-reported exposure to violence in their community was significantly associated with youth substance use in our study, results that are consistent with previous literature (23, 24). Our findings also contrast previous evidence among Latinx adolescents, suggesting that the most powerful of the three tested neighborhoods factors on substance use was negative neighborhood characteristics, rather than community violence (31). However, that study examined community violence at the neighborhood level —via crime rates—rather than individual-level perceptions—via youth-reported violence exposure, as done in the current study. One possible explanation is that youth using substances spend more time in areas where they are more likely to be exposed to neighborhood violence (61). Alternatively, it is possible that witnessing specific violent events in the neighborhood is a more significant risk factor for substance use than general community violence. The findings are also consistent with social disorganization theories (8), such that individuals in communities with low level social control may exhibit more delinquent and violent behaviors to exert informal control, which then leads to increased substance use risks. Lastly, the findings follow ecological theories and previous research suggesting that although the neighborhood environment contributes to substance use behavior, its effect is often less direct than proximal individual and interpersonal factors (20, 62). For example, neighborhood social cohesion has been shown to impact substance use not directly but through increased risk of depressive symptoms and exposure to peer substance use (39).

We found perceived negative neighborhood characteristics and collective efficacy to not be associated with youth substance use. Previous studies have found neighborhood factors to not impact substance use directly but instead exacerbate the relationship between exposure to violence and adolescent substance use (22). Thus, although community violence may place Latinx adolescents at greater risk for exposure to violence, it may be the individual psychological impact of that trauma exposure that more proximally predicts substance use. Despite this, there is evidence that neighborhood factors such as collective efficacy and social disorganization can impact substance use, moderating cultural fit among Latinx youth and parents, pointing to the nuanced role of these distal factors (42, 63).

Previous studies have shown that parents’ reports of social disorder were associated with increased substance use among Latinx and African American youth (31), and that conversely, positive perceptions of neighborhoods (e.g., greater sense of community) buffered against youth substance use risk (46, 47). However, contrary to our hypothesis, none of the parent-reported neighborhood-level factors were associated with increased or decreased substance use risk among the Mexican-origin youth in our study. The incidence of youths’ substance use was low in this sample, with 79% endorsing no substance use, which may have interfered with our ability to detect significant associations. In a manuscript published with this sample of Mexican-origin families, we found that parents and youth differentially reported on the same domains (e.g., parent reporting fewer youth mental health problems when compared to youth self-reports) (54). Thus, parents and youth may have differing perceptions of the same neighborhoods. Parent and youth discrepant reporting merits additional research, as it may mean that family members’ social networks within the same community may be vastly different. Future studies examining associations between parents’ perceptions of neighborhood-level factors and youth substance use should also consider examining subgroup risks (e.g., those at risk of maltreatment), and evaluate the protective roles of parenting (e.g., parent–child relationship quality) and cultural (e.g., ethnic identity) factors. Nonetheless, our results highlight the need to capture multiple neighborhood perspectives, even if individuals live in the same household, as different reports can differentially impact substance use outcomes.

While the current study has several strengths, including incorporating both youth and parent reports of multiple theoretically-informed indices of neighborhood characteristics that may influence youth substance use, some limitations should also be noted. As a cross-sectional study, the current results cannot inform on directionality or causality. However, they can draw attention to a relationship worth investigating. Future planned research with this sample aims to examine these risk processes over time with additional waves of data collection. Nonetheless, investigating risk processes related to the onset and early use of substances among youth is important for informing prevention strategies. Finally, while the current study represents a significant step forward in clarifying the role of community violence exposure, negative neighborhood characteristics, and neighborhood collective efficacy associated with youth substance use, we did not consider more complex models.

### Implications for policy, interventions, and research

Community violence remains a significant public health issue. Consequences include decreased wealth for families and more importantly, at the individual level, affected development across the lifetime (64). Interventions to address community violence at the policy level can include efforts to reduce structural violence through enacting policies and laws to reduce the social and economic marginalization that gives way to perpetual inequities (65). In addition, place-based interventions to address community violence are necessary because these are responsive to the target community. For example, South et al. (66) conducted a cluster randomized trial in predominantly low-income and Black neighborhoods. They found that the intervention of remediating abandoned houses was associated with reduced gun assaults and decreased weapons violations in those neighborhoods. Participation in an afterschool, park-based, mental health program, Fit2Lead, was associated with improved mental health outcomes in youth and parents a year later (67) and reduced youth arrests 2 years later (68). These results were from youth residing in urban areas with concentrated poverty where the majority of residents were Latinx or Black youth. These results provide strong support that local-based efforts in community settings can create a meaningful impact in reducing community violence and improving mental health overall. Our research sheds light on ways household members demonstrate differing perceptions of neighborhood characteristics, and these can impact youth health. More longitudinal data are needed to observe these changes over time, including when youth transitioning into young adulthood and when substance use is more likely to occur.

## Conclusion

Overall, the current study contributes significantly to the literature by highlighting the salience of youth-reported exposure to community violence as a neighborhood characteristic linked with substance use among Latinx youth. Policymakers, researchers, and community stakeholders can develop targeted interventions and prevention strategies by understanding the dynamics between parent and youth reports of neighborhood characteristics and substance use. Our results suggest that these strategies should focus on creating safe and inclusive neighborhood environments and reducing community violence, in particular. Additional strategies may involve improving community resources, enhancing social support networks, fostering open communication of neighborhood risks, and promoting collaborative efforts in addressing substance use behaviors.

## Data availability statement

The datasets presented in this article are not readily available because data collection is still ongoing and involves a racial and ethnic minoritized sample. The author/s are not able to release the data used in the current manuscript given the sensitivity of the data and their agreements with the Institutional Review Boards of the participating institutions. Requests to access the datasets should be directed to Sheri Markle, smarkle@mgh.harvard.edu.

## Ethics statement

The studies involving humans were approved by The University of Notre Dame Institutional Review Board, with cede reviews from participating sites. The studies were conducted in accordance with the local legislation and institutional requirements. Written informed consent for participation in this study was provided by the participants’ legal guardians/next of kin.

## Author contributions

JZ-D and MA contributed to the conception and design of the study. LZ conducted the analysis and methodology review. All authors contributed to the manuscript preparation, and read and approved the submitted version.
