# Supplementary material for: Mexican-origin parent and child reported neighborhood factors and youth substance use
Source: Front Psychiatry. 2023 Dec 1;14:1241002. doi: 10.3389/fpsyt.2023.1241002 (PMC10722282; doi:10.3389/fpsyt.2023.1241002)
Supplement: Supplementary file 1 [file Table_1.docx]

**Mexican-Origin Parent and Child Reported Neighborhood Factors and Youth Substance Use**

| **Supplementary Table 1. Association of neighborhood factors and youth substance use by Linear Regression models and Poisson models** | | | | | | | | |
| --- | --- | --- | --- | --- | --- | --- | --- | --- |
|  |  |  |  |  |  |  |  |  |
|  | Linear Regression - Youth | | | | Poisson - Youth | | | |
|  | Coff. | Std. Err. | 95% CI | *P* | Coff. | Std. Err. | 95% CI | *P* |
| Negative Neighborhood Characteristics | 0.101 | 0.06 | [-0.02, 0.22] | .112 | 0.017 | 0.01 | [0.00, 0.03] | .069 |
| Neighborhood Collective Efficacy | -0.021 | 0.06 | [-0.13, 0.09] | .713 | -0.016 | 0.02 | [-0.06, 0.03] | .502 |
| Exposure to Violence | 0.244 | 0.05 | [0.14, 0.35] | < .0001 | 0.141 | 0.03 | [0.08, 0.20] | < .0001 |
|  |  |  |  |  |  |  |  |  |
|  | Mother | | | | Poisson - Mother | | | |
|  | Coff. | Std. Err. | 95% CI | *P* | Coff. | Std. Err. | 95% CI | *P* |
| Negative Neighborhood Characteristics | -0.017 | 0.06 | [-0.13, 0.10] | .776 | -0.019 | 0.02 | [-0.06, 0.03] | .410 |
| Neighborhood Collective Efficacy | 0.007 | 0.06 | [-0.11, 0.13] | .901 | -0.003 | 0.02 | [-0.05, 0.04] | .891 |
| Exposure to Violence | -0.017 | 0.06 | [-0.13, 0.09] | .754 | -0.016 | 0.04 | [-0.09, 0.06] | .681 |
|  |  |  |  |  |  |  |  |  |
|  | Father | | | | Poisson - Father | | | |
|  | Coff. | Std. Err. | 95% CI | *P* | Coff. | Std. Err. | 95% CI | *P* |
| Negative Neighborhood Characteristics | -0.067 | 0.10 | [-0.26, 0.12] | .489 | -0.024 | 0.04 | [-0.10, 0.05] | .515 |
| Neighborhood Collective Efficacy | 0.053 | 0.09 | [-0.13, 0.24] | .577 | 0.028 | 0.04 | [-0.05, 0.10] | .467 |
| Exposure to Violence | -0.067 | 0.09 | [-0.25, 0.11] | .467 | -0.068 | 0.06 | [-0.18, 0.04] | .228 |
